# Key technical-tactical structures and anthropometric predictors of success in elite K1 kickboxing: a logistic regression based model

**DOI:** 10.3389/fspor.2026.1786929

**Published:** 2026-02-25

**Authors:** Dražen Čular, Matej Babić, Goran Jelaska, Igor Jelaska, Dino Belošević

**Affiliations:** 1 Faculty of Kinesiology, University of Split, Split, Croatia; 2 Einstein, Startup for Research, Development, Education, Trade and Services, Split, Croatia; 3 Virovitica County Hospital, Virovitica, Croatia

**Keywords:** athlete characteristics, competitive performance, match outcome, performance analysis, predictive modeling, technical efficiency

## Abstract

**Background:**

K1 kickboxing is an elite combat sport that combines striking techniques (punches, kicks, knee strikes) with tactical decision-making and anthropometric advantages. Success in K1 matches depends on both technical-tactical (TE-TA) structures and physical characteristics. However, few studies have developed comprehensive predictive models that integrate these factors to explain match outcomes in elite K1 competition.

**Objective:**

This study was to identify a model of success that incorporates key technical-tactical (TE-TA) structures and anthropometric characteristics that predict the outcome of kickboxing matches under K1 rules.

**Methods:**

A total of 96 qualifying matches for the K1 Final Grand Prix in Japan, comprising 192 fighter appearances from tournaments held between 1995 and 2012, were analyzed using logistic regression. Model fit was evaluated using the Hosmer-Lemeshow test, and discriminative power was assessed via Receiver Operating Characteristic (ROC) analysis.

**Results:**

The Hosmer-Lemeshow test (*χ*^2^ = 9.93, *p* > 0.05) indicated a good model fit, while the Receiver Operating Characteristic ROC analysis (area Under the Curve [AUC] = 0.84 demonstrated high discriminative power. The largest positive effects were observed for the rear-hand uppercut to the head, the lead-hand hook to the head, and the low rear-leg roundhouse kick to the lead leg, with each additional successful execution being associated with a 23.0%, 17.3%, and 12.6% increase in the odds of winning, respectively (e.g., OR = 1.23 for the rear-hand uppercut). Conversely, defensive structures involving hand blocks, whether against punches or leg strikes, were associated with 4.2% and 10.4% decreases in the odds of winning, respectively, suggesting these may be reactive structures taken under pressure. Additionally, each centimeter of greater height was associated with a 9.8% increase in the odds of winning.

**Conclusion:**

These findings provide expert coaches and scientists with guidance for optimizing contemporary training systems, enhancing tactical decision-making, and implementing predictive accuracy in elite-level kickboxing.

## 1 Introduction

Kickboxing is a combat sport that demands a combination of technical, tactical, and psychophysical abilities (1). K1 is a specific discipline within this sport, characterized by high-intensity combat that allows boxing punches, kicks, and knee strikes. Therefore, the outcome of matches depends on specific technical-tactical (TE-TA) structures and other anthropometric characteristics (2–4). Two domains, what the fighter does (TE-TA structures) and who the fighter is physically (anthropometry), are closely interconnected. Body morphology can influence both the selection and effectiveness of TE-TA structures (5). Moreover, in combat sports, researchers have demonstrated strong associations between motor and functional abilities and both TE-TA structures' effectiveness and time-motion analysis results up to 90% in some cases (6). Together, these findings suggest that performance results emerge from the interaction of TE-TA structures and an athlets physical attributes. Furthermore, the focus of sports science is often to define the characteristics that explain and form a model of performance (7). A higher frequency of certain TE-TA structures has been observed among more successful fighters (8, 9). The present study addresses this gap by developing a logistic regression model that incorporates both offensive and defensive TE-TA structures together with anthropometric variables, with the aim of estimating the likelihood of victory in high-level K1 bouts. This combined approach is important because it moves from describing successful fighters to predicting match outcomes based on modifiable TE-TA structures and relatively stable morphological characteristics, thereby providing coaches and analysts with a more powerful tool for performance profiling and decision-making in elite K1 kickboxing.

Beyond identifying the frequency and impact of these structures on wins and losses, it is necessary to develop a model that reliably characterizes a successful K1 kickboxer competing at a high level. Predictive and explanatory models have incorporated various components of biophysical characteristics in relation to athletes' success and quality (1, 10, 11). Our study proposes a prognostic-explanatory model that estimates a fighter's likelihood of success in high-level K1 kickboxing matches. This study includes a wide range of TE-TA structures and develops a logistic regression model specific to K1 kickboxing, an approach that has not been reported in earlier research at this level of detail. This research aims to contribute to a deeper understanding of the key determinants of success in elite-level kickboxing bouts and provide guidelines for optimizing training processes and managing fight tactics and prognostics.

Based on previous findings, we hypothesized that a higher frequency of certain offensive TE-TA structures would have a positive impact, and also, overreliance on defensive structures might lower the odds of winning. Anthropometric characteristics, especially height, were considered to be a quality for greater odds of success in fighting sports.

## 2 Methods

### 2.1 K1 kickboxing format and rules

K1 kickboxing is a high-intensity combat sport that integrates punches, kicks, and knee strikes, although clinching is strictly limited to a brief duration to execute a single knee strike. Standard bouts consist of three rounds lasting 3 min each, with a 1-min rest period between rounds. Prohibited actions include elbow strikes, throws, and strikes to the groin or back of the head. Victory is determined by knockout, technical knockout, or judge's decision based on a 10-point must system, where judges evaluate effective striking frequency, striking accuracy, defensive stability, and ring control. The main performance indicators analyzed in this study, including offensive and defensive TE-TA structures, strike frequency and accuracy, clinch effectiveness, and anthropometric variables, directly reflect the demands of this three-round format and the criteria by which elite K1 fighters are evaluated.

### 2.2 Sample of participants

This retrospective observational study, based on notational analysis of publicly available video recordings, included 96 matches, corresponding to 192 in-fight observations from 108 unique male athletes. All bouts were contested in the super heavyweight (open-weight) division, in qualifying tournaments for the final K-1 Grand Prix, spanning the period from 1995 to 2012. The years 2000, 2001, and 2011 were excluded due to the absence of tournaments. Overall, 24 matches ended with a knockout, 29 with a technical knockout, 41 by judges' decision, and 2 fighters were disqualified. All matches are publicly available online in multiple video formats on official and free-of-charge content platforms such as Daily Motion.

### 2.3 Ethics statement

The study was conducted in accordance with the Declaration of Helsinki. Ethical approval was granted by the Ethics Committee of the CAF—European Institute for Talents, Education, Research and Development (Approval No. CAF-EC-2016-03/EB). The study was conducted as a retrospective, non-interventional observational analysis based exclusively on publicly available video recordings, without any direct involvement of participants or collection of additional personal or sensitive data.

### 2.4 Variable sample

Based on a comprehensive review of existing literature and a consensus among expert kickboxing coaches and analysts, a predefined set of TE-TA structures was established for this study. The analysis encompassed 56 structures representing fundamental TE-TA structures, alongside two basic anthropometric characteristics (height and weight). A specific methodological innovation of this study was the dichotomous coding of strike effectiveness: a value of 0 was assigned to strikes that did not make contact with the opponent (technically valid but used as feints or for tactical setup), while a value of 1 was assigned to strikes that achieved contact (either blocked or landed directly). This approach enabled a more precise assessment of the tactical function of each strike.

Based on this detection method, 56 fundamental TE-TA structures were identified, of which 43 relate to striking techniques. Each strike was assessed at two levels of effectiveness, resulting in 99 distinct TE-TA structures (13 + 43 × 2), along with the two anthropometric structures. In total, more than 20,000 variable instances were recorded and included in the dataset. For statistical analysis, we identified and implemented all structures based on their frequency, strength, and impact. All structures with a variance inflation factor (VIF) below 10 were retained to avoid multicollinearity, and the final model was built using significant predictors, ensuring a robust and comprehensive analytical approach.

TE-TA structures were meticulously recorded through detailed video review and manually entered into text files (*.txt) following a strictly defined notational analysis protocol. This manual process ensured precise identification and quantification of all relevant structures.

Table 1 presents all the TE-TA structures detected in K1 kickboxing competitions.

**Table 1:** Defined K1 kickboxing technical-tactical structures.

Code	Description	Code	Description
*V*_1	Lead-hand straight punch to the head	*V*_29	Lead leg low roundhouse kick to opponent's rear leg
*V*_2	Rear-hand straight punch to the head	*V*_30	Rear leg low roundhouse kick to opponent's lead leg
*V*_3	Lead-hand straight punch to the body	*V*_31	Rear leg low roundhouse kick to opponent's rear leg
*V*_4	Rear-hand straight punch to the body	*V*_32	Lead leg mid roundhouse kick
*V*_5	Lead-hand uppercut to the head	*V*_33	Rear leg mid roundhouse kick
*V*_6	Rear-hand uppercut to the head	*V*_34	Lead leg high roundhouse kick
*V*_7	Lead-hand uppercut to the body	*V*_35	Rear leg high roundhouse kick
*V*_8	Rear-hand uppercut to the body	*V*_36	Lead leg side kick to the body
*V*_9	Lead-hand hook to the head	*V*_37	Rear leg side kick to the body
*V*_10	Rear-hand hook to the head	*V*_38	Lead leg side kick to the head
*V*_11	Lead-hand hook to the body	*V*_39	Rear leg side kick to the head
*V*_12	Rear-hand hook to the body	*V*_40	Spinning side kick to the body
*V*_13	Backfist strike	*V*_41	Spinning side kick to the head
*V*_14	Circular backfist strike	*V*_42	Spinning roundhouse kick to the head
*V*_15	Hand blocks against leg attacks	*V*_43	Spinning roundhouse kick to the body
*V*_16	Hand blocks against punches	*V*_44	Spinning roundhouse kick to the legs
*V*_17	Hand blocks against knee strikes	*V*_45	Lead leg knee strike to the head
*V*_18	Low block with lead leg	*V*_46	Rear leg knee strike to the head
*V*_19	Low block with rear leg	*V*_47	Lead leg knee strike to the body
*V*_20	High block with lead leg	*V*_48	Rear leg knee strike to the body
*V*_21	High block with rear leg	*V*_49	Lead leg knee strike to opponent's leg
*V*_22	Outer clinch and holds	*V*_50	Rear leg knee strike to opponent's leg
*V*_23	Inner clinch and holds	*V*_51	Lead leg scissor kick
*V*_24	Lead leg front kick to the head	*V*_52	Rear leg scissor kick
*V*_25	Rear leg front kick to the head	*V*_53	Left-side evasive slip
*V*_26	Lead leg front kick to the body	*V*_54	Right-side evasive slip
*V*_27	Rear leg front kick to the body	*V*_55	Left lean/shoulder roll
*V*_28	Lead leg low roundhouse kick to opponent's lead leg	*V*_56	Right lean/shoulder roll

Strike effectiveness coding is explained in the note below Table 2.

### 2.4 Measurement procedure

Match analysis was conducted through detailed video review, following a strictly defined notational analysis protocol. Before formal coding, three qualified raters participated in a standardized training session that explained the methodological approach to variable notation, operational definitions, and the step-by-step coding procedures; the training included theoretical instruction and pilot coding of a sample match to ensure consistency. After training, raters performed the notational analysis independently (blinded to one another) and were instructed not to consult each other during initial coding. Coded events were manually entered into text files (*.txt), consolidated into a data matrix, and analyzed with Statistica 12.0 (StatSoft, Tulsa, OK, USA). Inter-rater reliability was assessed on a random match sample (see Section 2.4 for ICC and correlation results).

### 2.5 Statistical analysis

Descriptive statistics were computed for the basic variables (mean, standard deviation, and confidence interval). Normality of distribution was assessed using the Shapiro–Wilk test, which indicated significant deviations from normality (*p* < 0.05) for the analyzed variables. Model fit was evaluated using the Hosmer-Lemeshow test (*p* > 0.05), and discriminative power was assessed via Receiver Operating Characteristic (ROC) analysis. For each variable included in the final model, odds ratios (OR) with 95% confidence intervals and logistic regression coefficients were calculated. The final predictive performance model was defined based on statistically significant predictors (*p* < 0.05).

Measurement reliability was assessed using the Intraclass Correlation Coefficient (ICC). The ICC was calculated using a two-way random-effects model with single measures and absolute agreement. Additionally, Pearson correlations between raters were used to assess inter-rater consistency (12). This procedure was conducted on a randomly selected bout sample, involving three qualified raters.

## 3 Results

As reported in the Methods, inter-rater reliability was confirmed [ICC(2,1) = 0.79; Pearson's *r* = 0.83–0.86], indicating good reliability (12).

Table 2 presents the basic descriptive statistics for all analyzed structures, mean ± SD, and 95% Confidence Interval (CI).

**Table 2:** Descriptive statistics for all analyzed structures: mean ± standard deviation and 95% confidence interval.

Code	Variable	Mean ± SD	95% CI
*V*_16	Hand blocks against punches	18.68 ± 22.54	15.47–21.89
*V*_1_1	Lead-hand straight punch to the head, contact made	11.81 ± 15.67	9.58–14.04
*V*_2_1	Rear-hand straight punch to the head, contact made	7.86 ± 9.04	6.58–9.15
*V*_22	Outer clinch and holds	7.73 ± 8.55	6.51–8.95
*V*_1_0	Lead-hand straight punch to the head, no contact	7.25 ± 8.18	6.09–8.41
*V*_30_1	Rear leg low roundhouse kick to opponent's lead leg, contact made	6.82 ± 7.72	5.72–7.92
*V*_9_1	Lead-hand hook to the head, contact made	6.82 ± 7.14	5.80–7.83
*V*_10_1	Rear-hand hook to the head, contact made	5.55 ± 7.89	4.42–6.67
*V*_15	Hand blocks against leg attacks	5.31 ± 7.16	4.29–6.33
*V*_2_0	Rear-hand straight punch to the head, no contact	4.09 ± 4.91	3.39–4.79
*V*_28_1	Lead leg low roundhouse kick to opponent's lead leg, contact made	3.83 ± 6.04	2.97–4.69
*V*_9_0	Lead-hand hook to the head, no contact	3.15 ± 3.89	2.60–3.71
*V*_32_1	Lead leg mid roundhouse kick, contact made	2.36 ± 4.26	1.75–2.97
*V*_10_0	Rear-hand hook to the head, no contact	2.30 ± 3.15	1.85–2.75
*V*_17	Hand blocks against knee strikes	2.04 ± 4.46	1.40–2.67
*V*_18	Low block with lead leg	1.79 ± 2.98	1.37–2.22
*V*_33_1	Rear leg mid roundhouse kick, contact made	1.71 ± 4.22	1.11–2.31
*V*_56	Right lean/shoulder roll	1.39 ± 3.18	0.94–1.84
*V*_48_1	Rear leg knee strike to the body, contact made	1.43 ± 3.05	0.99–1.86
*V*_6_1	Rear-hand uppercut to the head, contact made	1.30 ± 2.05	0.91–1.69
HT (cm)	Height (cm)	189.67 ± 7.48	188.61–190.74
WT (kg)	Weight (kg)	109.85 ± 17.12	107.42–112.29

Strike effectiveness: _0: No contact (feint or tactical execution); _1: Contact made (strike landed or blocked).

Among the analyzed technical-tactical structures, hand blocks against punches (*V*_16) stood out as the most prevalent defensive technique. The anthropometric data revealed considerable variability in fighter height and weight, which may contribute to diverse fighting styles and strategies within the super heavyweight division.

The model identified several significant predictors of victory. Positive effects per unit increase were found for successful strikes: rear-hand uppercuts to the head (*V*_6_1; 23.0% increase in the odds of winning), lead-hand hooks to the head (*V*_9_1; 17.3% increase in the odds of winning), and low rear-leg roundhouse kicks to the lead leg (*V*_30_1; 12.6% increase in the odds of winning). Negative predictors included defensive structures: each hand block against leg strikes (*V*_15; 10.4% decrease in the odds of winning), and each hand block against punches (*V*_16; 4.2% decrease in the odds of winning). Furthermore, fighter height exhibited a positive linear relationship with each additional centimetre, associated with a 9.8% increase in the odds of victory.

**Figure 1:**
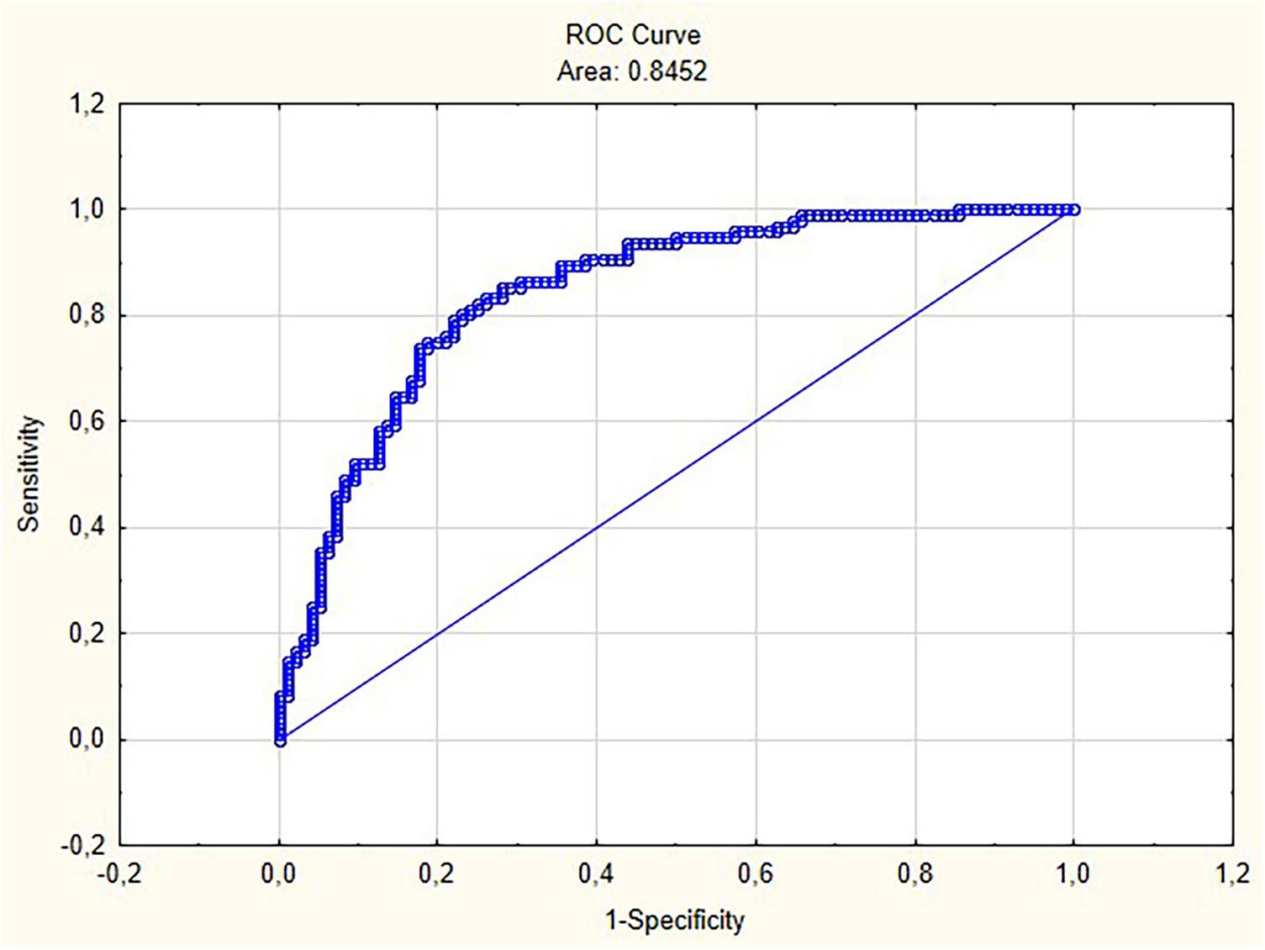
Receiver operating characteristic (ROC) curve.

The ROC analysis yielded an Area Under the Curve (AUC) of 0.84, indicating the model's high discriminative power in distinguishing between wins and losses (Figure 1). An AUC greater than 0.80 is considered strong (13). Furthermore, the Hosmer-Lemeshow test is pointing to a good model fit (*χ*^2^ = 9.93; *p* > 0.05). Table 3 displays the results of the binary logistic regression model, including coefficients, odds ratios, and significance levels for all predictors.

**Table 3:** Binary logistic regression model for predicting match outcome in elite K1 kickboxing.

Variable	*β* (Std. Err)	OR	95% CI	*p*
Hand blocks against punches	−0.04 (0.01)	0.95	0.93–0.98	<0.01
Lead-hand straight punch to the head with contact	−0.039 (0.02)	0.96	0.92–1.00	0.07
Rear-hand straight punch to the head with contact	0.05 (0.03)	1.05	0.98–1.12	0.13
Outer clinch and holds	−0.01 (0.02)	0.98	0.93–1.03	0.56
Lead-hand straight punch to the head without contact	−0.02 (0.03)	0.98	0.91–1.05	0.58
Rear leg low roundhouse kick to the opponent's lead leg with contact	0.11 (0.03)	1.12	1.04–1.20	<0.01
Lead-hand hook to the head with contact	0.16 (0.05)	1.17	1.06–1.29	<0.01
Rear-hand hook to the head with contact	0.05 (0.03)	1.05	0.97–1.13	0.18
Hand blocks against leg attacks	−0.10 (0.03)	0.89	0.83–0.96	<0.01
Rear-hand straight punch to the head without contact	0.06 (0.06)	1.06	0.94–1.20	0.28
Lead leg low roundhouse kick to the opponent's lead leg with contact	0.06 (0.04)	1.06	0.98–1.15	0.09
Lead-hand hook to the head without contact	−0.13 (0.07)	0.87	0.75–1.01	0.07
Lead leg mid roundhouse kick with contact	0.11 (0.06)	1.11	0.99–1.25	0.07
Rear-hand hook to the head without contact	−0.12 (0.09)	0.87	0.72–1.06	0.17
Hand blocks against knee strikes	−0.08 (0.06)	0.91	0.81–1.03	0.16
Low block with lead leg	−0.10 (0.06)	0.90	0.78–1.03	0.13
Rear leg mid roundhouse kick with contact	−0.04 (0.05)	0.95	0.85–1.06	0.38
Right lean/shoulder roll	0.06 (0.06)	1.06	0.93–1.21	0.32
Rear leg knee strike to the body with contact	−0.07 (0.09)	0.92	0.77–1.10	0.41
Rear-hand uppercut to the head with contact	0.20 (0.09)	1.23	1.03–1.46	<0.05
Height (cm)	0.09 (0.03)	1.09	1.02–1.17	<0.01
Weight (kg)	0.00 (0.01)	1.00	0.97–1.03	0.97

*β*, logistic regression coefficient; Std. Err, standard error; OR, odds ratio; 95% CI, 95% confidence interval for the odds ratio; p, statistical significance.

*p*-values are based on the Wald statistic.

### 3.1 Proposed mathematical model of success

This model provides coaches and analysts with a practical tool to estimate the impact of key technical-tactical structures and anthropometric factors on the likelihood of winning. It can guide training focus and tactical decisions to improve competitive performance.

The probability of winning (*P*) is calculated using the logistic regression equation: Linear predictor (*LP*) = *β*_0_ + *β*_1_*X*_1_ + *β*_2_*X*_2_ + .. + *β_n_X_n_*.

LP=−18.16−0.04×V16+0.11×V30_1+0.15×V9_1−0.10×V15+0.20×V6_1+0.09×Height

Index: *V*_16: Number of hand blocks against punches, *V*_30_1: Number of successful low rear-leg roundhouse kicks to the opponent's lead leg, *V*_9_1: Number of successful lead-hand hooks to the head, *V*_15: Number of hand blocks against leg attacks, *V*_6_1: Number of successful rear-hand uppercuts to the head, Height: Fighter's height in centimetres.

The probability of victory is then computed using the logistic function:

$$P=\frac{1}{1+e^{-LP}}$$

## 4 Discussion

This study identifies key technical, tactical, and anthropometric factors that are associated with winning or losing in K1 kickboxing matches. Descriptive statistics show a high degree of variability in both striking structures and anthropometric characteristics among super heavyweight fighters. Similar observations have been reported in prior studies focused on the morphological characteristics of super heavyweight kickboxers (10). Logistic regression identified several key structures significantly influencing the odds of victory or defeat. The model demonstrated strong discriminative power (AUC = 0.84), confirming its practical applicability. The rear-leg low roundhouse kick to the opponent's lead leg showed a strong positive association with winning. Each successful execution of this technique increased the odds of victory by 12.6%, which highlights the importance of low kicks in modern K1 kickboxing, not only as scoring techniques but also for impairing the opponent's mobility throughout a fight (2, 8). Similarly, precise punches, particularly the lead-hand hook to the head and the rear-hand uppercut to the head, proved to be strong success predictors. Each additional execution of these techniques increased the odds of winning by 17.3% and 23%, respectively. These findings suggest the need to develop advanced hand techniques and apply them effectively during critical moments of a fight (14, 15). Conversely, an increased number of hand blocks against punches and hand blocks against leg strikes was associated with decreased odds of victory by 4.2% and 10.4%, respectively. This suggests that excessive reliance on defensive strategies, particularly passive blocking, may be counterproductive. Such patterns may reflect a lack of initiative or tactical dominance, allowing the opponent to control the rhythm and flow of the fight. However, the negative association between frequent blocking and winning should be interpreted as associative rather than causal. High frequencies of hand blocks might reflect reactive behavior under sustained pressure, indicating that a fighter is in a disadvantageous position and forced into a defensive role. Thus, frequent blocking may be a consequence of being dominated rather than a direct cause of losing, highlighting the importance of maintaining offensive initiative to control the rhythm of the bout. These results are consistent with research emphasizing the importance of offensive engagement and tempo control (16). Among anthropometric characteristics, height emerged as a significant predictor of success. Each additional centimeter increased the odds of winning by 9.8%. Increase in winning odds per additional centimetre of height should, however, be interpreted with caution. Greater height is typically accompanied by longer reach, different body mass distribution, and potentially distinct tactical preferences, and those advantages are demonstrated in striking martial arts (1, 17, 18). Taller fighters may more easily maintain distance and exploit longer reach in both offense and defense. Nevertheless, it is important to note that technical proficiency can compensate for the lack of height, underscoring the importance of developing key TE-TA structures.

These findings have several practical implications for training. Modeling a successful kickboxer must include structures that reflect technical-tactical abilities, as well as training load that affects biomechanical and motor performance (19). Coaches and fighters should prioritize the development of effective low roundhouse kicks, precise head-level punches, and combinations involving these key techniques. Training should also emphasize proactive defense and minimizing passive blocking, with a strong focus on movement and positioning. While height may be an advantage in fighter selection, it should not overshadow the critical importance of TE-TA preparation. However, the retrospective design and the restriction of the sample to K1 rules and the absolute weight class can be defined as limitations that can shorten the generalizability of these findings to other combat sports and weight divisions. Despite the historical nature of the dataset, the analyzed bouts capture competitive patterns that are fundamental in contemporary K1 kickboxing, especially regarding fundamental TE-TA structures.

Although measurement reliability was confirmed through high ICC and Pearson correlations, the subjective nature of TE-TA annotation cannot be eliminated. Future research should incorporate larger and more diverse samples, automated match analysis tools, and explore the influence of psychosomatic and broader anthropological factors on match outcomes.

In summary, this study supports practical guidelines for training and tactical planning in elite K1 kickboxing, laying the groundwork for more advanced predictive modeling in combat sports.

Although the present findings are derived from elite-level competition, the applied modeling approach has relevance for understanding performance indicators across different stages of athlete development. Future research should extend such frameworks to longitudinal and comparative designs in order to examine how technical–tactical efficiency and anthropometric characteristics interact over time within developmental and talent development pathways.

## 5 Conclusion

The proposed model provides valuable insight into the technical-tactical structures and anthropometric characteristics of top-level K1 fighters. This study is distinguished by its analysis of a wide range of variables and the development of a new predictive performance model that influences match outcomes in K1 kickboxing. The model's high discriminative capacity (AUC = 0.84) confirms its reliability for predicting match outcomes and guiding the development of optimal fight strategies.

Notably, the model identifies a specific combination of techniques as low roundhouse kicks and precise head-level punches, as critical to success, while simultaneously highlighting the potential disadvantages of an excessively defensive approach.

The accumulation of successful techniques, such as a low rear-leg roundhouse kick to the opponent's lead leg, a lead-hand hook to the head, and a rear-hand uppercut to the head, along with greater height, significantly increased the odds of victory. Conversely, techniques like hand block against punches and hand block against leg strikes were associated with a greater risk of defeat. These findings can be used to adjust training and competitive strategies, allowing coaches and athletes to base their decisions on clearly defined predictors of success. This mathematical model can serve as a practical tool for this purpose.

## 6 Strengths and limitations

This study possesses several notable strengths, including the analysis of a relatively large sample of elite K1 bouts, the development of a novel predictive model with high discriminative power (AUC = 0.84), and the methodological innovation of dichotomous coding for strike effectiveness. The manual notational analysis, conducted with good inter-rater reliability, allowed for a granular examination of TE-TA structures. However, certain limitations must be acknowledged. The retrospective design and restriction to the K1 super heavyweight division limit the generalizability of findings to other rule sets, weight classes, or female athletes. The model's predictive accuracy (AUC = 0.84) was evaluated on the same dataset used for model development, without external or internal validation. Future studies should employ cross-validation techniques or independent test datasets to confirm generalizability. Although calculated reliability measures were strong, the inherent subjectivity of manual video analysis remains a constraint. Failure to account for within-fighter correlation may have resulted in underestimated standard errors and overstated statistical significance. While the direction and magnitude of effects are theoretically consistent with prior literature, the precision of estimates should be interpreted with caution. Among anthropometric characteristics, height emerged as a significant he analyzed TE-TA structures remain the fundamental core of contemporary K1 kickboxing. While tactical nuances and pacing may have evolved, the underlying biomechanical and strategic principles of effective striking and anthropometric advantages likely retain their relevance. Furthermore, while the model identifies associative predictors, the causal mechanisms linking frequent blocking to losing require further investigation (e.g., is blocking a cause or a consequence of being dominated?). Future research should employ prospective designs, incorporate automated tracking technologies, and integrate psychological and physiological variables to build more comprehensive performance models.

## Data Availability

The datasets generated and analyzed during the current study are publicly available in the Zenodo repository at https://doi.org/10.5281/zenodo.18075960.
